# Response to “Comment on cognitive function in the context of pharmacogenetic CYP2D6 variability and anticholinergic burden in older adults – results from the ActiFE study”

**DOI:** 10.1007/s00228-026-04183-8

**Published:** 2026-09-10

**Authors:** Linda Lorenz, Judith Berres, Michael D. Denkinger, Dietrich Rothenbacher, Stefanie Braig, Dhayana Dallmeier, Katja S. Just

**Affiliations:** 1 Institute of Clinical Pharmacology, University Hospital RWTH Aachen, Aachen, Germany; 2 Institute for Geriatric Research, Ulm University Medical Centre, Ulm, Germany; 3 Geriatric Centre Ulm at AGAPLESION Bethesda Hospital, Ulm, Germany; 4 Institute of Epidemiology and Medical Biometry, Ulm University, Ulm, Germany; 5 Department of Epidemiology, Boston University School of Public Health, Boston, MA USA

**Keywords:** Older adults, Anticholinergic burden, CYP2D6, Pharmacogenetics, Cognitive impairment

## Abstract

We clarify that our analyses were exploratory and hypothesis-generating, with systematic stratification across CYP2D6 metaboliser groups rather than selection based on an interaction *p* < 0.2 threshold. A post-hoc Benjamini–Hochberg sensitivity analysis provided supportive, but not confirmatory, evidence for the hypothesised stronger association between anticholinergic burden and cognitive function among individuals with reduced CYP2D6 activity. Given the limited subgroup sizes, ceiling effects, and generally high cognitive performance of the cohort, these findings should be considered hypothesis-generating.

Dear Editor,

We like to thank the authors for their constructive comments on our study examining the association between cognition and anticholinergic burden in the context of pharmacogenetic CYP2D6 variability [1, 2].

It is true that the absence of a formal multiplicity correction entails a risk of false-positive findings. We explicitly considered our analyses exploratory and hypothesis-generating and did not interpret the observed associations as confirmatory evidence [1].

We would like to clarify the characterization of the *p* < 0.2 threshold as an exploratory criterion for assessing potential effect modification. This threshold was not used to determine which metaboliser subgroups were carried forward into analyses. Stratified analyses were conducted systematically for all metaboliser groups with sufficient sample size.

To further address the concern regarding multiplicity, we performed a post-hoc sensitivity analysis using the Benjamini–Hochberg (BH) procedure for the six interaction tests from the fully adjusted model (Model 4) of the primary analysis and separately for the corresponding six interaction tests in the secondary analysis [1]. The results are summarised in Table 1. In the primary analysis, the IM × ABS_2D6_ interaction at the 75th percentile remained below the BH-adjusted significance threshold corresponding to a false discovery rate (FDR) of 10% and 20%, but not 5% (raw *p* = 0.012; BH-adjusted *p* = 0.072). In the secondary analysis, the corresponding interactions at the 50th and 75th percentiles had BH-adjusted p-values of 0.066 and likewise remained below an FDR threshold of 10%.

**Table 1:** Results of Benjamini–Hochberg (BH) false-discovery-rate (FDR) sensitivity analysis

Primary analysis						Secondary analysis				
	Raw *p*-value	BH-adjusted *p*-value	FDR 5%	FDR 10%	FDR 20%	Raw *p*-value	BH-adjusted *p*-value	FDR 5%	FDR 10%	FDR 20%
IM x ABS_2D6_, Q25	0.455	0.634	no	no	no	0.884	0.941	no	no	no
IM x ABS_2D6_, Q50	0.052	0.156	no	no	**yes**	0.022	0.066	no	**yes**	**yes**
IM x ABS_2D6_, Q75	0.012	0.072	no	**yes**	**yes**	0.022	0.066	no	**yes**	**yes**
PM x ABS_2D6_, Q25	0.827	0.827	no	no	no	0.941	0.941	no	no	no
PM x ABS_2D6_, Q50	0.331	0.634	no	no	no	0.128	0.256	no	no	no
PM x ABS_2D6_, Q75	0.317	0.634	no	no	no	0.355	0.533	no	no	no

*IM* intermediate metaboliser, *PM* poor metaboliser, *ABS* anticholinergic burden score deriving from CYP2D6 substrates, *Q* quartile

Importantly, as published, we did not regard an interaction p-value < 0.2 as sufficient evidence of effect modification on its own [1]. Greater interpretative weight was given to patterns in which the interaction signal was accompanied by effect estimates in the corresponding stratified analyses, that were consistent in direction and magnitude with the research hypothesis, while considering their confidence intervals and precision. The effect estimates remained compatible with the hypothesized pattern of a stronger association with reduced CYP2D6 activity, including for IMs at the 50th percentile. Given the limited subgroup sizes, however, we regard this consistency as supportive rather than confirmatory evidence.

More generally, the analyses were guided by the biologically motivated hypothesis stated in our introduction that the association between cognitive function and anticholinergic burden would be most pronounced in individuals with genetically reduced CYP2D6 activity. This focus on reduced CYP2D6 activity was defined prior to data analysis, although no single primary comparison was formally preregistered.

Regarding the clinical interpretability of our findings, we agree that this is limited because the strongest finding was observed at the upper end of the MMSE score distribution. However, at the 50th percentile of the MMSE score distribution, the association among IMs showed a comparable consistent negative direction. Due to a ceiling effect and the small number of participants with possible cognitive impairment, the absence of findings at the 25th percentile does not allow to conclude that such an association does not exist among individuals with lower cognitive performance. Notably, given the generally high level of cognitive performance in the cohort, the 25th percentile should not be equated with a subgroup of participants with clinically meaningful cognitive impairment. Accordingly, our findings do not allow conclusions regarding the magnitude of this association in individuals with more pronounced cognitive impairment.

The present findings do not identify a specific group of older adults who would currently benefit from individualized anticholinergic risk assessment. Rather, our translational conclusion is intended as a hypothesis for future research. If the observed effect modification by CYP2D6 metaboliser status is confirmed in larger and more cognitively heterogeneous populations, preferably using longitudinal designs, CYP2D6 metaboliser status might represent one additional factor contributing to individualised risk assessment.

## Data Availability

The datasets analysed during the current study are not publicly available due to the conditions provided in the informed consent at the time of recruitment but are available from the corresponding author on reasonable request.
